# Taking pills for developmental ails in Southern Brazil: The biologization of adolescence?

**DOI:** 10.1016/j.socscimed.2014.11.028

**Published:** 2015-10

**Authors:** Dominique P. Béhague

**Affiliations:** a Vanderbilt University, USA; b King's College London, UK; c London School of Hygiene and Tropical Medicine, UK

**Keywords:** Brazil, Child psychiatry, Pharmaceuticals, Psychoanalysis, Epistemology, Prototype, Medicalization

## Abstract

In the late 1990s researchers in Pelotas Southern Brazil began documenting what they considered to be unacceptably high rates of licensed psychotropic use among individuals of all ages, including youth. This came as a surprise, since the vast majority of psychiatrists in Pelotas draw on psychoanalytic theory and approach pharmaceutical use, especially for children and adolescents, in a consciously tempered way. Drawing from a longitudinal ethnographic sub-study, part of a larger 1982 birth cohort study, this paper follows the circuitous trajectories of emergent pharma-patterns among “shantytown” youth over a ten-year period, exploring the thickly layered and often moralized contingencies in which psychodynamic psychiatrists' intention to resist excessive pharmaceuticalization both succeed and crumble. I juxtapose these trajectories with the growing salience of an “anti-biologizing” explanatory framework that psychiatrists and researchers are using to pre-empt the kind of diagnostics-driven “biopsychiatrization” so prevalent in North America. My analysis suggests that psychiatrists' use of this framework ironically contributes to their failed attempts to “resist” pharmaceuticalization.

## Highlights

• Biopsychiatric logics are often implicated in the global rise of psychotropic-use.
• In Brazil, psychotropic-use prevails despite therapists' resistance to such logics.
• This resistance obscures the moral and social contingencies of pharmaceuticals.
• Attempts to resist biopsychiatric logics ironically contribute to psychotropic-use.

## 1 Introduction

In the 1990s researchers in Pelotas, Southern Brazil, began documenting what they considered to be unacceptably high rates of psychotropic use among individuals of all ages (Rodrigues et al., 2006). For many, this was unsettling news. Data suggested that much of this use resulted from prescriptions written by psychiatrists, yet the vast majority of psychiatrists in Pelotas are psychodynamic in orientation and though they have been prescribing medications since the 1950s, they have always done so in a consciously tempered and temporary way, subservient to the deeper work of psychodynamic therapy. Elevated levels of psychotropic medication-use among children and youth came as a particular surprise, since Pelotense child psychiatrists rely on the works of Heinz Kohut and Donald Winnicott, amongst others, for whom environment and sociality are therapeutically central. The impetus to be cautious about psychotropic drugs has only grown since Brazil's de-institutionalization movement of the 1990s. Rejecting both the elitism of “pure” psychoanalysis and bioneurological models of the brain, Pelotense psychiatrists have worked hard to create a re-invigorated, decentralized, and democratic social psychiatry.

How might one analyze this paradox? Are therapists saying one thing and doing another? Are patients and parents demanding pharmaceuticals in ways that challenge therapists' ideals? Perhaps all are being subtly persuaded by larger forces: the globalization of diagnostic manuals, bioscience, the market, and industry?

These are the questions that Pelotense psychiatrists and researchers are themselves beginning to ask. Referring to international literature concerned with the globalization of Anglophone biopsychiatry (e.g. Watters, 2010), many are concerned that a rapidly globalizing and highly-profitable pharmaceuticalized North American model of the brain will soon pervade and that psychodynamic orientations will in turn subside. I was often told, for example, that newly emerging diagnoses in biopsychiatry such as depression and attention-deficit disorder (ADD) are “socially constructed” symbols of Anglo neuro-psychiatry, canonized in diagnostic manuals and backed by industry. Or, even more powerfully, that therapists who are unable to “resist pharmaceuticalization,” prescribing when unnecessary or failing to transition patients off medications, are unwilling pawns of the broader industry-infused globalization of biopsychiatric ways of reasoning.

It would be tempting to adopt my interlocutors' interpretative framework and assume that will only be a matter of time before biopsychiatry comes to dominate over dwindling socially-psychodynamics orientations. Yet psychiatrists' emphasis on a biologizing episteme as the key modality through which professionals and patients are persuaded to prescribe and use pharmaceuticals seemed misplaced. On the ground – in everyday life, in clinics, in formal interviews – the language of the brain and biological immutability rarely surfaced spontaneously or in any sustained way. And, as scholars have shown (and my interlocutors frequently acknowledged), psychodynamic theories are not impervious to pharmaceutical reductions (Metzl, 2003), nor does pharmaceuticalization always proceed through biologizing logics (Kitanaka, 2012; Lakoff, 2006). There is clearly more at play in pharmaceuticalization and more at stake, also, in the rise of an anti-biologizing anti-pharma episteme.

In this paper, I follow the circuitous trajectories of emergent pharma-practices among “shantytown” youth over a ten-year period, exploring the thickly layered contingencies through which psychodynamic psychiatrists' intention to resist excessive pharmaceuticalization both succeed and crumble. I juxtapose these contingencies with the way Pelotense therapists variously construct knowledge about the diverse therapeutic trajectories they observe and help to produce. Of the various ways of knowing that are at play, I give specific attention the increasingly salience of an anti-biopsychiatric episteme. Why is this episteme compelling if biopsychiatric logics are not pervasive? How does it seep into and transform clinical and social life, shaping lives of therapists and patients alike? And what other ways of knowing does it obscure from view?

My answer to these questions points to the interplay of two epistemic modalities for understanding pill-taking – the (rationalist) explanatory model and the (morally-infused) prototype. I explore how these modalities become entangled with therapeutic practices, mental states, and life-course trajectories. Among the many consequences produced by this entanglement is this one: reliance on explanatory models of how biopsychiatric logics hold sway (or can be resisted) diverts attention from the broader moral, social, structural, and economic contingencies that drive (or circumvent) pharmaceuticalization. This reliance paradoxically contributes to psychiatrists' failed attempts to “resist” pharmaceuticalization, thus helping to produce an emergent biotherapeutic form.

## 2 Methods

I draw empirical material from long-term (1997–2007) fieldwork with an array of experts (*N* = 92), including therapists, school staff, local government officials, those involved in grass-roots movements, and with a sample of 96 young people and their families. These young people were selected at random from a pool of participants interviewed in the 1997 survey of the 1982 Pelotas birth cohort study, a prospective ongoing study of 5914 children (Victora et al., 2003). Random sampling was used not because we intended to conduct probabilistic analyses, but because we sought to capture a full array of life-course experiences, including those of particularly introverted and socially isolated youth.

Using participant observation and repeated semi-structured and informal interviewing with youth, their mothers and other key family members and friends, our research was conducted over a decade in the lives of these youths, from the time they were 15 to their 26th birthdays (from 1997/98–2007/08). Fieldwork was conducted by myself, another anthropologist, and four research assistants (see Béhague et al., 2008; Victora et al., 2003 for methodological and analytical details). Ethics approval was obtained from the Federal University of Pelotas' Faculty of Medicine ethics board at each new follow-up; informed consent was elicited from participants at each of these. When cohort children were under 18 years of age, informed consent was obtained from parents and children; once over 18 years of age, informed consent was obtained only from cohort youth.

## 3 Theorizing the social life of ways of knowing

In the early 1980s, Allan Young called attention to the theoretical limitations of the “explanatory model” approach for understanding how patients' make sense of their illnesses (Young, 1982a). The explanatory model was originally proposed by Arthur Kleinman in the 1970s as a framework for use in both research and the clinical encounter, and it continues to be widely used, especially for promoting cultural sensitivity in the clinic. Young argued that explanatory models, though useful pedagogically, are rationalist forms of knowledge premised on linear logics and causal propositions. Because explanatory models presuppose that the classification of etiology, symptoms, and treatment is a central feature in all ways of knowing, they fail to recognize the myriad and non-linear ways people produce knowledge about health and illness (Young, 1982a).

Young's argument was initially built upon empirical work with ‘lay’ knowledge systems in which cause-and-effect logics are not always central defining characteristics. But he and other scholars have also pointed to the ways rationalist assumptions can skew our understandings of how biomedicine becomes persuasive and authoritative (Lock et al., 2000; Young, 1980). This argument is a more difficult one to make, and may appear counter-intuitive, for biomedicine's unparalleled power rests precisely on its “rationality”: the search for clear codification and causal relationship, the operational value of simplification, and the lure of quick fixes (Good, 1994). Indeed, researchers have consistently underscored the way simplifying theories of brain disorders, used in highly effective ways by industry, constitute the key mechanism through which widespread acceptance of specific diagnostic categories and associated psychotropic medications have proliferated (Conrad and Bergey, 2014; Timimi, 2005).

Yet I want to argue that a great deal of social science research on biomedicine privileges its *bioepistemic* powers, over and above other forces at play. In Pelotas, I am not convinced that “resistance” to “bioepistemic” rationales actually accounts for the tempered use of pharmaceuticals that psychodynamic psychiatrists strive for, nor do I think that bioepistemic rationales are core to the recent rise in psychotropic use. Yet this is precisely the story – an explanatory model – that has gained circulation globally (e.g. Watters, 2010), and it is the story that Pelotense therapists and experts are beginning to endorse as their own. This explanatory model can be put succinctly thus: the notion that brain disorders are caused by underlying biological–neurological phenomenon and can be treated with pharmaceuticals underpins widespread acceptance of and desire for pill-taking. Within this model is the converse notion: namely, if more complex understanding of suffering linked to mind, person and society are retained, all would see the pill for it is: a bioreductionist quick fix with potentially long-term negative effects. I will call this an explanatory model of bioepistemic authority.

This explanatory model is not merely a theoretical abstraction. It has a social life and is in this sense ‘operative.’ Succinct and persuasive in its etiological attributions, its retelling creates a unifying, provocative, and stabilizing call-to-action (Löwy, 1988). As I became attentive to the contexts in which an explanatory model of bioepistemic authority is elicited, I noticed that therapists, teachers, and at times parents and youth, bring this model into focus at key moments: at the frustrating limits of what social psychiatry can do to rectify deep suffering; in enthusiastic instances of psychodynamic vindication; and most importantly, in response to the *moral* discomforts of other less visible ways of knowing that are linked to moral judgments on what pharmaceutics are, do and mean. Importantly, I show that these judgments are made against the backdrop of intertwined ideas about class, poverty and analytical capacity.

These morally-infused and informal ways of knowing are akin to Young's notion of the “prototype.” Prototypes are stories of sickness episodes that people have witnessed or learned about and they are used to frame subsequent episodes, producing knowledge in disorderly non-causal ways (Young, 1982a,b). I began to think about prototypes when I noticed that the kinds of therapeutic languages and practices used by therapist, parents, and patients varied depending on patients' socio-economic status and age. Broadly put, a psychodynamic language of “adolescent” crisis and transformation resonated more intensely in the mid to late teen years and among upper-middle class and upwardly mobile youth. In contrast, a medical language of behavior and containment, with hypothesized diagnoses of attention and conduct problems, appeared more readily for youth in lower socio-economic status and at younger ages. Over time, these two patterns developed into different kinds of psychotropic use, one more psychodynamic, temporary and transformative, and the other more stagnating, chronic and prone to biotheorizing. Of the 96 youth in the ethnographic sample, 19 were prescribed a psychotropic medication at some point in their life; of these, 6 used medications in a temporary way, as therapists intend, whereas 13 entered into what therapists themselves identified as an unproductive and stagnating medicated therapeutic trajectory. As overarching patterns, these two prototypes proved to be salient in epidemiological analyses of the larger 1982 cohort samples as well (Béhague, 2004).

In what follows, I introduce two young women, Juliane and Rosane, whose life experiences I take to be representative of the varied forces that go into the making of these two patterned prototypes. I take these prototypes to be indicative of two contrasting “kinds” of people (Hacking, 1995) or “person-making” regimes (Martin, 2006). Though reproduced in contemporary forms of self-making, they hark back to late 19th/early 20th century Romantic notions of the “elite” psyche, a Freudian aesthetic that juxtaposes upper class intellectualism against the “retrograde” psyche of the underclass (Duarte, 1999–2000). Most psychiatrists, aware of the problematic ethical legacies of this history, claim this aesthetic to have been largely overcome. Yet I will demonstrate that these two prototypes are very much alive, and that they come to life in therapeutic routines as “ethical exemplars” (Laidlaw, 2013). And here is the crux of my argument: when therapists sense the moral discomfort these exemplars produce, they revert to a simpler explanatory model hinging on the powers of bioepistemic authority.

## 4 The “storm and stress” benchmark

For Juliane, the end of childhood and beginning of adolescence began at age 13 not with menstruation and first boyfriends, but with the death of her father, Emilio. Feelings of mourning and betrayal intensified when Juliane's family discovered that Emilio had a second “wife” and three children in a neighboring city. Juliane's family, already living well under the poverty in one of Pelotas' newest shanties, underwent rapid downward mobility and Vera, Juliane's mother, unable to make ends meet, was forced to send her children to live with extended family members for weeks at a time. Vera's recollections of these early years were both blurred and strikingly concise. Juliane began spending more time “on the streets,” at times for days on end. Illicit drug-use became part of her everyday experience, as did exposure to street violence and sexual encounters she did not always want. At age 16, she began having “visions” and talking of “wanting it all to go away.” Vera explained that during her “crises,” Juliane would “talk incoherently and wander anxiously through the house, believing it haunted by spirits.” Vera had long been taking her children to a Kardecist center and so she sought help from a Spiritist healer, but Juliane improved only temporarily. During another crisis, Vera rushed her daughter to Pelotas' one remaining psychiatric hospital. The attending psychiatrist admitted Juliane but then discharged her a few hours later, telling her to follow-up at her local clinic. Though Vera made an appointment, Juliane failed to attend.

Juliane had not abandoned school and eventually Juliane's teacher took her to visit the school psychologist, Lizette. Like the vast majority of young people referred to a school psychologist, Juliane was highly skeptical; she viewed teachers, school staff, and therapists as inherently elitist and thus only superficially caring. Yet Lizette's focus on Juliane's justifiably difficult “adolescent turmoil” intrigued Juliane. Adolescence is always turbulent, Lizette explained, and Juliane's would be more protracted than most, given her life circumstances. Yet it would entail not just anxiety and sorrow, but the acquisition of important abilities – analytical, reflective, social, and pragmatic. The notion of adolescence as agency-inducing comforted Juliane, as did its de-stigmatizing framing. She returned to therapy and Lizette began to involve Juliane's teacher and mother in the process. “The psychologist would tell my mother to just let me be,” Juliane recalled, “and not criticize [when I was acting strange]. Once the psychologist asked me why I no longer cried as much. I told her, ‘because father told me to stop. He is taking care of me. He isn't here, but he tells me I must go to school. If not he will get angry with me.’ Well, when I told my mother this, we were walking down the street holding hands. She looked at me with those big eyes, her jaw dropped, and she let go of my hand.”

The normalization of Juliane's experiences was linked to her appreciation of the “democratizing” nature of the clinical encounter. Juliane told me, with some surprise, how Lizette actually listened and refrained from simply telling her “how to behave” as teachers so often did. Juliane explained that she enjoyed talking to Lizette because it showed her that the two of them weren't “all that different,” even though Lizette was clearly middle class. Lizette, in turn, reflected on how Juliane had a spark, a drive for change and upward mobility that she did not frequently encounter, making her an ideal candidate for in-depth therapy. Underpinning these interpretations was a subtly moralized linking of the analytical capacity for psychological transformation with the avid desire for upward mobility – a moral-pedagogic stance that would gain force in the context of Juliane's future encounters with a psychiatrist.

Several elements were critical to Juliane's initial productive engagement with therapy: clinical democratization, normalization of symptoms, concerted effort to work with school and home, and recognition of Juliane's difficult life circumstances. Both Juliane and Lizette made explicit reference to these elements as they planned next therapeutic steps. Yet when I asked Lizette in a formal interview about her somewhat unusual success with Juliane, she gave me a response that I subsequently heard in meetings and interviews time and again – a response that I identified to be an explanatory model focused on the power of a psychodynamic interpretation to resist facile bioreductionism, to inculcate a desire for something more than medication. “We have a problem with the medicalization of education,” she explained, “Everyone, even teachers are taking pills. What happens is you become more distanced from affect. Medicine becomes a *mordaça* (gag, muzzle). Juliane is different. She is learning to look her emotions in the eye.” The work of therapy, she continued, is not a simple fix; the patient needs to be shown the value of a slower therapeutic pace.

The pace was indeed slow and the transformation that Juliane eventually experienced emerged only after many setbacks. There were long periods of time when Juliane withdrew from school and from visiting Lizette, and in her 17th year, during yet another crisis, Vera took Juliane to hospital where she was given a referral to a public sector psychiatrist. This time, Juliane was more accepting and she met with the psychiatrist, Luiz. Upon discovering Juliane's suicidal thoughts, Luiz prescribed an anti-depressant. Like Lizette, he foregrounded not diagnosis or medication but rather the experience of adolescence as a transitional state. He told Juliane that the medication was a temporary measure and that they would focus on broad-ranging “incremental” rehabilitation. Juliane feared the anti-depressants' potential side effects and worried they might be addictive. Yet her desire for much-needed respite outweighed these concerns. “It might be the only way to get better. It will give me some time,” she explained. She initiated a medicated psychodynamic journey with the optimistic notion that the end of adolescence would bring the end of medication and reduction in distress.

The fluid and normalizing conceptualization of adolescence that both Luiz and Lizette referred to recalls psychodynamic-psychologist Stanley Hall's early 20th century theory of adolescent “storm-and-stress,” one that sanctions a slow and protected developmental pace. Relative to his contemporaries, Hall ascribed to a more optimistic Lamarckian interpretation of evolutionary theory; and he was the first to argue that a child's “developmental” period ran well into “adolescence” and that adolescence constituted a key “metamorphic” phase created through the clash of “primitive” (child-like) and “civilized” (adult) psychologies. Contemporary permutations of this theory see adolescence as a “moratorium,” a period of non-committal experimentation with self and society (Cairns, 1998).

As I observed Juliane move toward a “Hallesque” transformation, I noticed how “the social” became a source of knowledge for theorizing cause and seeking therapeutic improvement. Luiz understood Juliane to be caught in a “dialectic” between societal (and class) conflicts and internal adolescent conflict. He, like others, explicitly referred to self-psychology theorists, such as Heinz Kohut and Erik Erikson, who made their canonical mark by exploring how the social acts in and through the individual. As one of Luiz’ colleagues stated, “The emergency of our current social angst, the transition to democracy, a history of dictatorships, is placed *into* the adolescent. This complicates what is already a turbulent life phase.” Like others, Luiz conceptualized adolescence as a unique opportunity for broader social change. “We all say the subject lives in a depressive [dysfunctional] society, and we leave it at that. Throw [our] hands up (shrugs shoulders). Well, if that's the case, then we need even more to *not* medicate. We must investigate the person's life struggles, and overcome these.”

For Juliane, the idea that her problems were related to the harshness of the social world rather than to her specific psychological or cognitive “failings” was enthusiastically received. Luiz himself never presumed that Juliane's situation would improve only through her own analytic work. Like Lizette, he worked with Juliane's teachers and mother, and he encouraged Juliane to seek support and make concrete changes. For Juliane, this included, quite centrally, an intensification of her “Spiritist studies” and increased participation in her local shantytown neighborhood association, where she helped with several initiatives, from canvassing the neighborhood to preparing for the next bingo game. As she herself indicated, religion and activism gave her a sense of social belonging and it helped her in school, for she learned how to engage in her life without “thinking too much” and by focusing on “smaller steps.”

The social also seeped into the clinic via Luiz's democratizing ethos. He did not exempt himself – a member of the upper-middle class – from scrutiny and considered “transference” dynamics to include the social conflicts and class-distinctions that young people such as Juliane experience on a daily basis. Juliane, in turn, welcomed the legitimacy this bestowed. “I was worried they [psychologist and psychiatrist] would just tell me what to do,” she explained, “what was wrong with me … but he talked to me like I was any other person.” For Luiz, this democratizing attitude helped to increase empathy. Yet the lessening of class differentials also created an opportunity for the socialization of Juliane into a middle class value-system; it drew Juliane into a different world view and allowed her to see that not all members of the upper-class are disparaging. In humanizing Luiz, her desire for upward mobility grew, as did her desire for Luiz's *moral-pedagogic* support in seeking that mobility.

James Laidlaw's discussion of the role of “exemplars” in ethical life is eminently useful here. He argues that moral life is shaped not simply by rules and rationalized obligations. Rather, “individuals cultivate themselves as ethical subjects in relation to chosen exemplars; [that is] historical heroes or living people they interact with and chose as ‘teacher’ in their own personal development” (Laidlaw, 2013: 83). Exemplars mediate between fact and values, between what is and what should be (p. 85); they are pedagogic prototypes. In Luiz Juliane saw an exemplar. As her situation improved, Luiz, in turn, looked upon her as his exemplar, a prototype of the ‘good’ patient qualities that allow psychodynamic techniques to work. Indeed, when comparing patients, Luiz, like Lizette before him, highlighted those unique personal-moral qualities that made Juliane “capable” of insight. “Adolescents [like her],” he explained, elicit a greater sense of promise because they come with “fewer defenses …. Reflection is very painful, [but it is] at the heart of development for the adolescent.” Youth who were deemed capable of transforming this pain into adolescent-infused “opportunity” tended to embrace rather than critique the drive for upward mobility (as many shantytown youth do); this fact alone underscores that what became central in Juliane's emerging clinical form was not epistemic faith in anti-biologizing psychodynamic analysis, but rather entrenched social–ethical hierarchies.

It was in the context of this morally-charged enactment of adolescence that stopping medication became as important – if not more important – than the sanctioned reprieve it represented: no longer just a therapeutic aim, it became a moral act. The impending “end” of adolescence troubled Juliane. “I'm not *too* worried about the [adult] responsibilities I have to take on,” she told me, “but I feel anxious about losing my adolescence, about not taking full advantage of it.” Advantage how, I asked? Juliane spoke about wanting to feel free to be a “crazy” adolescent. But she also worried that she might not be able, adult-enough, to stop her medication, to wean herself of the therapeutic supports she now had in place. How would she know if her adolescence was coming to an end and what if she failed? Though fearing failure, the moral imperative to build strength and well-being during adolescence became increasingly salient. This therapeutic framing was starkly reaffirmed when Juliane explained that one key risk of psychotropic-use was the slippery slope of addiction and entrenched destitution. “These medications aren't so different from street drugs. Whether its sleeping pills, anti-depressants or street stuff, your life can easily degrade.”

Juliane eventually proved herself to be capable. Her incremental trajectory to a more stable situation continued, and Juliane and Luiz decided it was time she transition into talk therapy only. Increased participation in social life, in her Spiritist studies and her neighborhood association, and an ability to “speak openly” with friends and family were identified as key signs of her readiness to withdraw medication. Luiz began to see her more intermittently while Lizette continued seeing her in the school setting. “With time,” Juliane recalled, “I was able to talk to the psychologist, who knows a ton about me. This was more important than any medication. Now when I need to talk, I let it loose!” That Juliane could legitimately “extend” her adolescence when needing some respite also resonated powerfully: “When those crazy adolescent emotions attack me, I go to see [Lizette]. Poor thing (laughing), she sits there as I talk, and I joke with her [pretending to be her therapist]. I tell her, ‘I bet you're dying for me to give you a clean bill of health.’ She jokes back, ‘I'm the one that's dying to give *you* a clean bill of health!’”

The playful inversion of the therapist–patient relationship in this exchange demonstrates just how potent Lizette's “exemplar” role became in shaping the pedagogic prototype that Juliane came to personify. By age 18, Juliane's crises had largely abated. Luiz continued encouraging her to engage in multi-institutional sources of support. By the time Juliane turned 19 she had stopped medication completely, held a part-time job, had a close circle of friends, was near to completing her secondary education, and had become engaged to a young man with a high school degree and a good job. Two years later, she and her fiancé migrated away from their parental homes and began living in a flat, away from their natal shanty community.

Juliane's life-trajectory was unique but by no means singular. The visible minority of women who shared her experiences are indicative of an emerging social trend. Most overcame their struggles in large part because education, civic organization, religion, family, *and* transformative psychodynamics converged to create an upwardly mobile “adolescent” form of personhood. Their achievements in education alone were clearly exceptional vis-à-vis the norm for those with similar socio-economic backgrounds. Seeped in an aura of optimism, a few went further, entering college to pursue careers in psychology, social work, and teaching – professions where helping others to “develop” were identified as markers of their own psychodynamic success.

That Juliane's transformation was as structurally supported as it was internal was evident to Juliane, Lizette, Luiz, her mother, and her teachers. Though all had nurtured a therapeutically meaningful adolescent identity, and though this identity had at times been imbued with ethical judgments on which “kinds of people” are deemed capable of achieving it, rarely had it been divorced from the social contexts that enabled it to emerge. This was set to change.

Throughout her early 20s, Juliane reconstructed her life narrative to focus quite centrally on her maturation, on how she had managed to “resolve her adolescence,” conquer her identity, achieve a legitimate place in society, and avoid any sort of dependency, be it chemical or inter-personal. “The anti-depressant was passing for me,” she stated, “it just helps a bit … Today, I know how to be calmer, to have a more tranquil attitude. I think, I reflect. What do I want, what do I not want. Not being able to do that characterizes the infantile side of adolescence.” She rarely recalled her Spiritist studies or neighborhood association activities, the institutional supports she had received in school, her growing friendships, or the direct effects of having a more stable income. Instead, she produced a classic explanatory framework centered on the transformative power of deep self-fashioning to successfully “resist” bioreductionist pharmaceuticalization.

In moments of reflection more distanced from actual case-histories, therapists also produced similar narrative reconstructions. Luiz, for example, when summing up Juliane's case in a formal interview, relied on an explanatory framework that made causal inferences about the over-riding therapeutic power of reflective capacity – of the appreciation for deep analysis and the perils of medication. Yet for both him and Juliane, reverberating behind the surface meaning of this explanatory model was the middle-class ethical exemplar that had been in the making all along, a morally charged prototype of the *type* of patient who is psychological *capable* of withdrawing medication. Poignantly, in one of my last visits, Juliane off-handedly compared her life-trajectory with those of some of her childhood peers whose lives “on the streets” had led to near-total school truancy, joblessness, and dependency on both prescription and illegal drugs.

This self-vindicating knowledge-form recalls classic critiques of conservative forms of psychoanalysis. Fernando Duarte has argued that psychodynamic traditions in Brazil are saturated in Weberian instrumentalism, one in which Freudian-inspired ideologies became integral to the elite's “civilizing” mission of the early 20th century and to the reproduction of class distinctions (Duarte, 1999–2000: 156). Not only are certain kinds of person-types being recreated, but young women's gravitation to “self-making” professions such as teaching, psychology, and social work reproduces the person-making apparatus. Dominic Boyer argues that unlike industrial labor, products of contemporary “mental labor” are inextricably bound to personhood, since producers of mental labor have themselves to be produced if their “commodities” are to be given a distinctive form (Boyer, 2005: 251). Similarly, Juliane and her therapists became embroiled in a type of mental labor that produced both future producers of mental labor *and* a broader compelling justification for their work – that is, resistance to pharmaceuticalization.

## 5 Arrested adolescent development

The ironies of Juliane's storm-and-stress development are particular stark when contrasted with the lives of youth at the other end of the spectrum. Though a developmental notion of “adolescence” has become so commonplace that virtually all youth hoped to “realize” it, within a year or two of initiating adolescence, most low-income youth were quick to point out that poverty and lack of opportunity – rather than “psychological development” – lay at the heart of their difficulties. Referrals to therapy were thus prone to sensitive politics of blame, as feelings of undue culpability mired therapeutic encounters. What then happens to therapeutic journeys, and to the production of knowledge about pharmaceuticals, in the absence of a Hallesque “storm-and-stress” adolescence?

Abject poverty and repeated migration marked Rosane's childhood and teen years. Rosane recalled one of her parents' moves as particularly unsettling. Precipitated by rumors that the local government held plans to level all illegal squatter homes in their community, they salvaged as many belongings as they could carry and moved to Rosane's aunt's home, situated in one of Pelotas's most removed peri-urban settlements. The nearest school was a 45-min walk away. Rosane, then 13, and her younger siblings did their best to settle into new classes of unknown peers run by, as Rosane once described, ill-humored teachers. In an effort to help their parents, she began skipping class to work *biscates* (informal petty jobs) and over the ensuing year, she found herself “giving up” in school, failing exams and getting into fights with classmates. In her 14th year, her teacher sent her to the school psychologist, highlighting *problemas de conduta* (conduct problems) and *falta de attençao* (lack of attention). As with Juliane, the school psychologist underscored the tempered and normative even “protective” qualities of adolescence, if only Rosane could attend school more regularly and be given some tranquil time at home. The psychologist invited Rosane to return the week after and sent a note home suggesting Rosane's mother, Fernanda, make an appointment at her local clinic with the attending psychiatrist.

From the upstart, the Hallesque notion of adolescence that the psychologist introduced flew in the face of the deprivation Rosane felt continuously undermined her efforts. “Adolescence? Ha! They talk about adolescence,” she once retorted, “that doesn't exist. I'm 14, but it will never exist for me.” Though Rosane complied with the request that she visit the psychologist again, she was dismissive, insinuating that classist sentiments had been at the heart of her teacher's referral. “It's not fair,” she said, “they always peg *us*. All this talk they want, it's just to tell us what to do, what is wrong with ‘us’ [shantytown] kids.” That she had approached her adolescence with hope made the therapeutic encounter all the more bitter.

Fernanda empathized with Rosane's sentiments, and often echoed her daughter's strong sense of social injustice. Yet Rosane's state continued to worsen and because Fernanda no longer knew how to respond to her daughter's frequent outbursts, crying episodes, and screaming attacks, she sought help at her local clinic. The attending physician explained that Rosane was externalizing her emotions in a way that presented as conduct problems and inattention only because her immaturity still precluded her ability to articulate and process her troubles. Acknowledging the family's difficult home life and the oft-tense school environments that youth must endure, he did not want to resort to an ADHD diagnosis and explained that Rosane's behaviors signaled underlying (but temporary) anxiety and depression, a natural response to difficult situations. He prescribed an anti-depressant, explained the medication to be a stop-gap, and asked Rosane and Fernanda to return in two weeks' time for a “proper in-depth analysis” of her situation with the clinic psychiatrist.

At a conceptual level, this physician used both a socially sensitive stance, similar to that Luiz and Lizette had employed, and an anti-biologizing ethos. Yet in the absence of the more immediately pragmatic, engaged and multi-pronged approach that seems to have made a difference for Juliane, Rosane's situation worsened. She took the medication for only a week, never returned to the clinic, and her rejection of all things psychological increased. She told me that her problems related not to depression or “immaturity” but to the converse. “I haven't had an adolescence,” she said, “school failed me … all that I have experienced – it made me grow up too quickly.” Even so, she remained in school for the better part of her 15th and 16th years, and not so infrequently, her teachers would send her to the school psychologist for “misbehavior” or “lack of concentration.” According to Rosane, these sessions usually lasted a quarter of an hour and consisted of misplaced questions to which Rosane responded in short “yes's” and “no's.”

As Rosane's emotional responses grew more strained, a new knowledge-form gained visibility: a loosely articulated morally-imbued prototype that I understood to be the converse of what Juliane's life came to represent. Rosane told me that her teachers “just keep telling me I'm acting childish” and that the psychologist made reference to her “disorganized” development. The psychologist, in turn, told me that Rosane, unable or unwilling to engage in deep analysis, was prone to the dangerous medicated patchwork that was becoming so common in schools. Fernanda herself began using developmental language to compel her daughter to action: “Rosane's adolescent explosions are still there. She still wants to be a child … but it is time for her to face up.” Rosane's own self-reflections also changed, as she internalized these developmental notions, and began speaking of weepiness, adolescent introversion, even identity confusion. “Now I see what they meant when they talked about my adolescence,” she said, referring to her early teen-hood, “I was such a *besta* (beast) back then, without conscience. I felt like an adolescent, my emotions were all over the place and no one, including myself, seemed to know when it would stop. Maybe I should have stuck to the medications.”

When I asked the school psychologist to expand on Rosane's pathological “desire for childhood,” she explained that when different “lines” of development – cognitive, emotional, sexual – evolve incoherently, some more quickly than others, a truncated form of adolescence emerges, rife with anxiety and recalcitrant behaviors. Several psychiatrists subsequently identified this theory to be part of the conservative Freudian school of ego-psychology established by Anna Freud and Heinz Hartman, amongst others. Though most claimed this “decontextualized one-person theory of development” to have fallen out of favor in Pelotas, it was precisely this framing, a prototype – the converse of all that Juliane's “civilizing” therapeutic trajectory achieved – that took hold in Rosane's life. As Rosane's rejections of social injustice increased so did interpretations of her childish (immature) quality become entrenched, as did the burgeoning sense that perhaps she would never be capable of adult-like introspection. Thus, by default to a historically-entrenched knowledge-form rather than by canonical intention, adolescence came to signal not normative “storm and stress,” but rather the failure of the unfolding ego.

Frustrated by life, including these depictions of her character, Rosane disengaged completely. I found it increasingly difficult to find her at home. At first, Fernanda made excuses for Rosane's absence, but then told me in a shameful and saddened tone that Rosane had “taken to the streets.” In 1999, a then 17 year old, Rosane began dating a young man in his early twenties, a renowned drug user and trafficker. When I caught up with her later that year, she recounted how her life with him had “taken over,” how she contented herself with her newfound friends, a group of *marginais* [“vagabonds”], as she called them. She began experimenting with marijuana and cocaine, and soon left school altogether. Her street-life exposed her to levels of violence that most parents shelter their children from through strict curfews. Within a few months, Rosane was robbed and assaulted, and then witnessed a friend embroiled in the drug trade die. In the two years that ensued, Rosane withdrew, spending days at a time either in her room or on the streets. When she “looked at herself,” she said she saw a life that had digressed “beyond repair.”

In her 18th year, Fernanda took Rosane to the psychiatric hospital. Like Juliane, she was given a referral for outpatient psychiatric care. Unlike Juliane, Rosane never followed up. Instead, she walked away feeling shattered: “The hospital took me in [for a bit] but they said this was perhaps a passing adolescent thing … The doctor told me to ‘go home, stay home, and sleep.’ So I did … These things happen … But until it passed, I could not look at anyone in the eye.” She waited. At times, Rosane wondered if perhaps the notion of “arrested development” she had encountered at the local clinic, in school, and in her relationship with her mother, might be true. In 2000, nearing her 19th birthday, she found herself pregnant with an unwanted child and in an unwanted relationship. She began living with her then-boyfriend, Beto, the child's father, hoping to turn a new leaf, at times excited for motherhood. A few months before she gave birth, Beto died mysteriously. Though Rosane managed to secure a regular part-time job as a cleaner in the evenings when her grandmother could care for her baby, her weepiness and exhaustion mounted.

At this near-tipping point, Rosane went to her local primary care clinic. The attending psychologist referred her to a public sector psychiatrist, Paulo. Given the severity of her symptoms and suicidal thinking, he prescribed an anti-depressant. In good socially sensitive psychodynamic fashion, Paulo explained medication to be a temporary facilitator for the more important social-analytical work at hand. Rosane welcomed this thought – it indicated, once again, that her state might be transitional. Paulo was the first to recognize that Rosane's life circumstances did not lend themselves to an “adolescent awakening,” yet he felt committed to supporting, with whatever structural means possible, the sense of youthful developmental opportunity he had successfully enabled for other patients.

Even so, the creation of transformative socio-personal analysis was thwarted almost from the start. Paulo's attempts to encourage Rosane to “think and reflect,” to demonstrate that strong sense of injustice he knew so many shantytown youth rightfully have, fell flat. Instead, exhaustion took over, interspersed with what Rosane herself had begun to identify as “adolescent-like fits” of anger. Rosane recounted how therapy would help her “work hard” to accept adult responsibilities, to resolve the “stresses” of adolescence: “Adulthood comes when you stop the craziness, when you can wake up and *ligar* (turn yourself on). Otherwise, you enter into depression and that whole thing.” In an almost mournful way – perhaps for a lost childhood and adolescence – she sought in therapy the cultivation of self and social acceptance. “I am better,” she reflected, in her 21st year, “but I still feel irresponsible, young … (pause). Everything that happened to me had a good side, because it made me say to myself – I'm going to stop this madness (pause). I have lost too much time. It will pass.” Waiting became the bare-minimum she could muster.

In reflecting on this situation, Paulo himself explained that patients such as Rosane can easily enter into a “medicated holding pattern” – justified by therapists' impetus to avoid further deterioration, but grossly under-problematized. This interpretation, though spoken about in the third person, was notably reflexive. It was precisely at this therapeutic crossroads – one in which the severity of Rosane's situation collided with Paulo's disquieting critical sensibilities – that a bioepistemic explanatory framework emerged indirectly and jointly with a biologizing trajectory itself:

Though Rosane and Paulo had avoided clear-cut diagnosis, Rosane's language became more focused on *symptoms* of a potential *disorder*, rather than, as a social psychiatrists might want, *contingent* states of mind that *respond* to difficult circumstances: her stomach aches, dizzy spells, overheated sensations, and “fits” became critical sources of information. Rosane wondered if she “had something.” Somewhat reluctantly, Paulo began to analyze these bodily symptoms by making reference to a psychoanalytic theory of “somatization” in which physical ailments are seen as idioms of the psyche. Paulo's reticence was warranted. Though a focus on the body could in theory be helpful, like ego-psychology, theories of somatization are recognized in Pelotas as prone to an elitist separation of mind over body. Indeed, this framing did not help Rosane, as she took it to mean that her problems had worsened *because of* her inability to recognize their psychological origins.

Rosane's sense that “something [physiological] was there” became compounded when she attempted to wean herself off her anti-depressant. Though following Paulo's advice, each time she tried, she felt worse and each time she interpreted this to be proof of her “condition.” Her lack of success then blurred the distinction between symptom, side-effect, and withdrawal effect, producing a new “post-pill” context for the production of knowledge. Here, Rosane began to use a clearer diagnostic label – depression – alongside a more biomedical immutable interpretation of her predicaments. “My depression,” she stated, now 22 years old, “It appeared for the first time in adolescence and came back with the hypertension. I had to get back onto the anti-depressants. I'm trying to stop again, but I notice it immediately when I stop, something is there. I'm not sure it will ever go away.”

Paulo was reticent to accept Rosane's speculative biotheorizing. He had a niggling sense that in her case, prolonged medication-use had dulled her, worsened her symptoms. And the search for an alternative therapeutic trajectory did not seem – and indeed was not – completely unrealistic (see Béhague, 2009 for a description of these alternatives). Her life situation had actually improved somewhat; with full support from her mother, she was managing to sustain a regular work schedule while still caring for her daughter.

At other times, however, Paulo showed heightened sensitivity to the gravity of Rosane's situation. He wondered about her capacity for talk-therapy, suggesting that perhaps the multigenerational weight of social, economic and personal hardship made deep analysis futile, perhaps counter-productive. Yet even in this carefully posited statement, uncertainty resounded. Though he did not articulate it as such, he recognized the slippery slope of classism here; the historic force of the converse of Juliane's “civilizing” psychoanalytic exemplar; the diminishing status of the “primitive” prototype incapable of deep analysis and thus prone to somatization, to accepting a pharmaceutical *mordaça* (muzzle).

It was precisely at this juncture that Paulo's position began to shift. Seeking a less morally charged account of what he now considered to be Rosane's pharmaceuticalized therapeutic stagnation, he appealed to a more clearly articulated explanatory framework focused on the causal epistemic powers of biopsychiatry. Perhaps, he speculated, Rosane was wishing for a quick fix. He himself had used the term “depression” quite loosely and he continued making reference to her socially-induced “nerves,” as she once had. Perhaps therapy was not evolving into something more, as it had for others patients he had known, because she was somehow convinced of the indisputably bodily and biological nature of the constellation of symptoms that beset her. Perhaps the media had somehow persuaded her to adopt the more immutable notion of depression to which she now ascribed. These explanations provided Paulo with greater epistemic certainty and a specific course of action, one that seeks to convince patients (and colleagues) that psychological phenomenon are complex, that the pill is a temporary stop-gap.

This is precisely what Paulo then attempted. Losing sight of the broader social and institutional constellations that accounted for Rosane's therapeutic stagnation (and for the therapeutic transformations that Paulo, like Luiz, had enabled in patients such as Juliane), he focused on re-socializing Rosane to appreciate a non-biologizing episteme. Depression is just a rough category, he told her. The pill is just one step to other changes; it need not indicate a static “condition.” Rosane listened and dutifully expressed a desire for a deeper understanding of her suffering's origins and for a life off medication. Yet even then she used corporeal terms to imagine how talk-therapy might help her to “expel the stress and loosen the body.” In her 23rd year, she resigned herself to the chronic markings of an adulthood struck by depression: “I know people who take several medications regularly,” she said “one for nerves, one to sleep, another for depression. It's complicated. I suppose we all will arrive at the phase of having to take those *remedinhos* (little medicines) all the time.” Rosane's position vindicated Paulo's “anti-biologizing” position, and through repeated micro-iterations such as these, an explanatory framework of bioepistemic authority solidified.

This bioepistemic explanatory framework can be conceptualized, following Foucault's work on discursive formations, to be an emerging episteme, a space of dispersion and displacement (Burchell et al., 1991: 55). In public forums of meetings and workshops I observed Paulo himself adopt distinctly formulated explanations for the rise of psycho-pharmaceuticals in his city. “Today,” Paulo said in one such meeting, “the failure of psychodynamics arises largely from the demand for a quick fix, on everyone's part. This demand is the result of giving the pharmaceutical industry free reign to ‘name’ a ‘disease’ through the marketing of a specific drug.” This explanatory position became increasingly evident throughout the years of my fieldwork, especially as therapists extracted themselves from the specificities of their patients' lives and debated the issues in public meetings or responded to my questions in formal interviews. Similar rationales have appeared with growing frequency in publications concerned with the globalizing force of pharmaceuticalization in Brazil. This theorizing, while familiar, even comforting to my anthropological sensibilities, also surprised me. Though offered “critically,” it struck me as abrasive in its dismissal of the structural complexities and long-standing moralizing prototypes at play in both the therapeutic “successes” and over-medicated “failures” I and my interlocutors witnessed.

## 6 Conclusion

The narrative reconstructions that Juliane, Rosane, and their therapists ended with – narratives of the resilient powers of psychoanalytic self-making on the one hand and of the reductionist powers of bioepistemic authority on the other – represent an explanatory model that many in the social sciences share. It would be inaccurate to argue that it is “false.” Biopsychiatric epistemologies are indeed emerging in Pelotas, if only, at this point, through indirect routes, outside the realm of clearly orchestrated intentional action. Patients do at times “demand” drugs and diagnostic rigidity, especially once chronicity of medication-use sets in; and therapists at times find relief in providing their patients with a simplifying bioepistemic narrative. My interlocutors are right to raise awareness about biopsychiatric simplifications.

Yet this explanatory model *is* glaringly partial. It is partial at the very basic level of the explicit articulations that people produce. And, even if the language of the chemical brain (or some bio-equivalent) were more prevalent in Pelotas, as it may become, there are clear indications that this explanatory model would still fail to adequately reflect empirical reality. Simply put, the attribution of pharmaceuticalization to the *epistemic* dimensions biopsychiatry, first and foremost, fails to account for forces at play that people don't mention explicitly – social, moral and economic forces that have prepared the ground for pharmaceuticalization much prior to the post 1980s re-biologization of psychiatry. As I have shown, judgments about pill-taking – when and if to take them, how long to take them for, what it *means* to take them and to get off of them – are made in the context of powerful and long-standing morally-infused prototypes, or person-making projects. Importantly, this explanatory model also fails to account for the role of economics, structure, pragmatism and patience, the complex multi-institutional support systems that enable success stories such as Juliane's. As I have described elsewhere, some of these success stories occurred in the context of deep commitment to a consideration of ‘the social’ as both etiologically and therapeutically central, and they did not *always* engender a vindicating anti-biologizing psychodynamic posture (Béhague, 2009).

The explanatory model of bioepistemic authority is not just full of gaps. It is also an enactment; it has a social life; its use actively *displaces*. Explanatory models displace by using “desocializing concepts” – e.g. “bioepistemic authority” or “resistance to biopsychiatric ways of reasoning” – that are linked to a simplified and easily shared vocabulary that holds intelligible “surface meaning” (Young, 1982b). “The power to displace,” Emily Martin writes, “[emerges by] means of a reduction in scale” and the “mobility” that is enabled through miniaturization (Martin, 2006: 282–283). The (social scientific) explanatory model of bioepistemic authority is also a miniaturizing one: it simplifies understanding; uses a vocabulary that is easy to share; and circulates rapidly, traveling the globe in tandem with pharmaceuticalization itself.

What are the specific moments in which the impetus to displace arises? I have shown that therapists and patients turn to this explanatory model in moments of moral vindication (Juliane) and moral discomfort (Rosane). Further, I would argue the narrative of psychodynamic vindication and an explanatory model of bioepistemic authority are being co-constructed precisely *because* of the moral discomfort that cases such as Rosane elicited. Recall that Rosane's therapeutic paralysis was lamented; and that Paulo's designation of Rosane as a “therapeutic failure” signaled the hope – and even imperative – for another way out. Medication is creeping in precisely when therapists experienced profound crises, as they opened their clinics and minds to the suffering of shantytown youth with a level of intimacy not yet seen in the history of Brazilian psychiatry. Thus, I would argue, therapists' attraction to an explanatory model of bioepistemic authority increases in tandem with the need for the medicating act to become a social anesthetic.

These displacing, or anesthetizing, powers have ontological effects that are important to recognize. By limiting therapeutic action, the focus on bioepistemic authority – and psychodynamic “resistance” to it – renders therapists and patients passive, more distanced from complex ways of knowing, and from the conditions that exacerbate or improve distressed lives. Thus, the very use of this explanatory model must be included as one among other social, economic, moral forces accounting for the emergence of chronic and stagnating biotherapeutic forms, the very kind of “local biology” (Lock, 1995) therapists wish to avoid. This is not a gratuitous critique, for this same Pelotense world provides us with exemplary moments in which the impetus for a personally and socially transformative therapeutic form is realized, often against considerable odds (Béhague, 2009). And, though Pelotas appears to be comprised of a unique epistemic tradition, it is very likely that even in corners of the world where biopsychiatry is more entrenched, a polarizing for-or-against positionality vis-à-vis biopsychiatry also conceals broader ethical challenges at stake in pharmaceuticalization. What might be possible if we stared these challenges and insecurities in the eye (Kleinman, 2006), rather than hide behind a discourse of anti-biologization (Rose, 2013)? How can we become more attentive to the ways pharmaceutical practices become linked to different value systems and not just different epistemic rationales? Might we then be able to formalize the structural and moral dimensions of the lasting therapeutic transformations that do occur in Pelotas, and elsewhere? As these questions indicate, I believe it is only by tackling the historically entrenched ethical challenges described in this paper that I believe social psychiatry will realize its full potential.
